# Exploring the spatiotemporal drivers of malaria elimination in Europe

**DOI:** 10.1186/s12936-016-1175-z

**Published:** 2016-03-04

**Authors:** Xia Zhao, David L. Smith, Andrew J. Tatem

**Affiliations:** WorldPop project, Department of Geography and Environment, University of Southampton, Highfield, Southampton, UK; Fogarty International Center, National Institutes of Health, Bethesda, USA; Department of Zoology, University of Oxford, Oxford, UK; Flowminder Foundation, Stockholm, Sweden

**Keywords:** Malaria elimination, Europe, GIS, Malaria risk mapping

## Abstract

**Background:**

Europe once had widespread malaria, but today it is free from endemic transmission. Changing land use, agricultural practices, housing quality, urbanization, climate change, and improved healthcare are among the many factors thought to have played a role in the declines of malaria seen, but their effects and relative contributions have rarely been quantified.

**Methods:**

Spatial datasets on changes in climate, wealth, life expectancy, urbanization, and land use trends over the past century were combined with datasets depicting the reduction in malaria transmission across 31 European countries, and the relationships were explored. Moreover, the conditions in current malaria-eliminating countries were compared with those in Europe at the time of declining transmission and elimination to assess similarities.

**Results/conclusions:**

Indicators relating to socio-economic improvements such as wealth, life expectancy and urbanization were strongly correlated with the decline of malaria in Europe, whereas those describing climatic and land use changes showed weaker relationships. Present-day malaria-elimination countries have now arrived at similar socio-economic indicator levels as European countries at the time malaria elimination was achieved, offering hope for achievement of sustainable elimination.

**Electronic supplementary material:**

The online version of this article (doi:10.1186/s12936-016-1175-z) contains supplementary material, which is available to authorized users.

## Background

The global range and intensity of malaria transmission has greatly reduced since the beginning of the last century [1, 2]. Nowadays, there are still 97 countries and territories with ongoing malaria transmission [3] and 35 of them are aiming to eliminate the disease [4]. In contrast to this, post-elimination countries tend to display a ‘sticky’ state of malaria elimination stability [5, 6], despite malaria vectors continuing to exist, large numbers of imported malaria cases and no control measures in place. With global malaria eradication back on the international agenda [7, 8] and increasing investment and efforts focused towards regional elimination, understanding which factors have played a significant role in the decline of malaria in post-elimination countries and continue to play a part in limiting risks of re-establishment can provide valuable information to countries aiming to rid themselves of the disease.

**Fig. 1:**
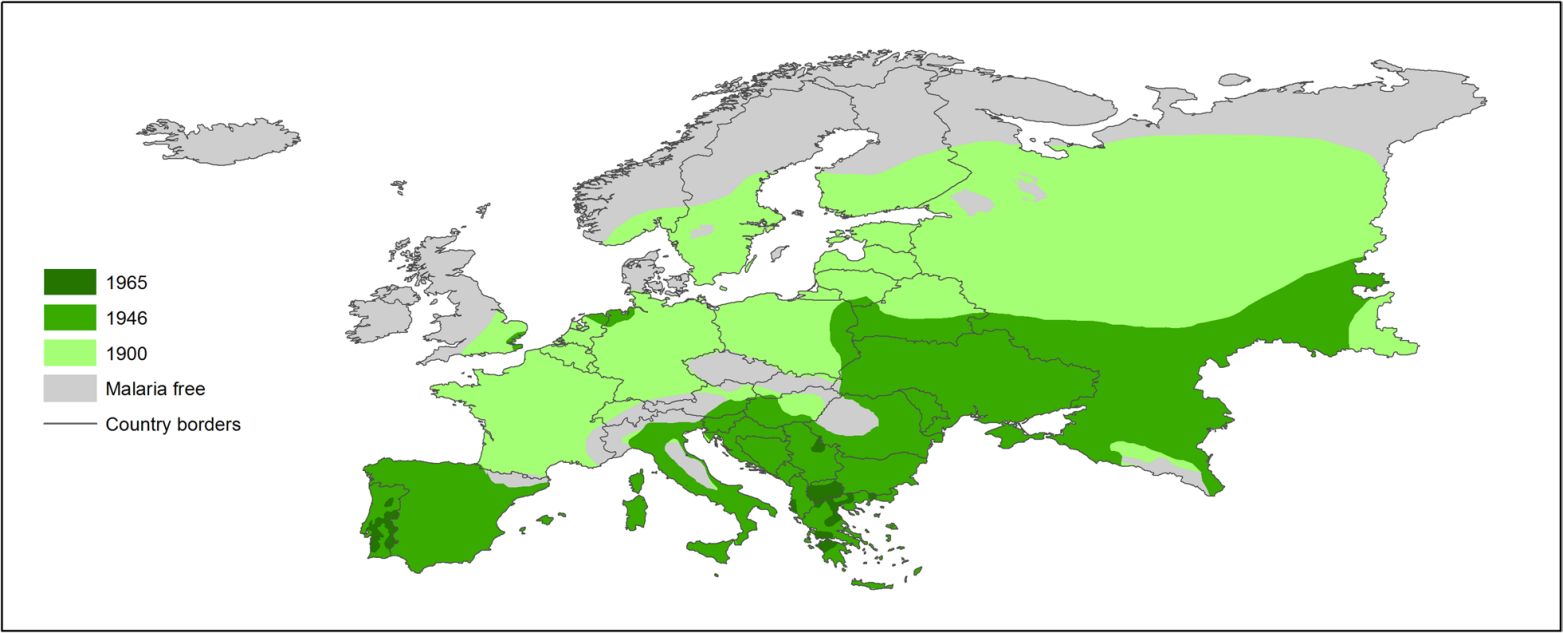
Malaria distribution in Europe for the ~1900–1965 period. All-cause malaria infections were included and areas with high and low malaria risk were merged. Present-day country borders are shown. *Source* Hay et al. [1]

**Fig. 2:**
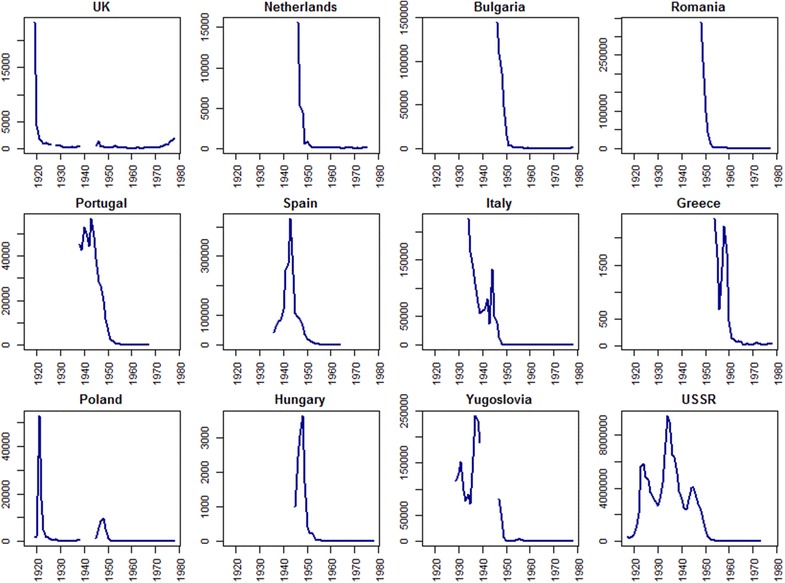
Reported malaria case numbers in European countries from 1917 to 1978. The *blue lines* represent the reported all-cause malaria case numbers. Source: Bruce-Chwatt and de Zulueta [9]. Yugoslavia [52] and the Union of Soviet Socialist Republics (USSR) [53] were existing countries during the 1917–1980s and 1922–1991, but both became sets of independent countries. Yugoslavia was composed of Croatia, Macedonia, Montenegro, Slovenia, Bosnia and Herzegovina, and Serbia; USSR included Russian Federation, Ukraine, Latvia, Lithuania, Belarus, Estonia, and Moldova

Malaria was once prevalent in almost every country in Europe. Considerable shrinkage of the range of transmission began from the early 20th century in the northern part of Europe, progressing to the southern edge (Fig. 1). At the same time, the intensity of transmission declined, except for certain outbreaks related to the First and Second World Wars [9] (Fig. 2, data collected from Bruce-Chwatt and de Zulueta [9]). By 1975, Europe was declared free of endemic malaria transmission and has retained that status ever since.

Factors that might have driven the decline of malaria in Europe have been widely discussed. In general, these can be classified into two categories: factors related to an early ‘natural’ disappearance [10] and later, declines accelerated through the addition of human interventions [11–13]. Specifically, in the early 20th century, when human efforts to control malaria were limited or non-existent, a spontaneous decline of malaria occurred in many regions of mainland Europe [10], with different factors speculated to be drivers. For example, in France and Denmark, vectors were found to have changed their biting preferences from humans to domestic animals, probably due to the warmer environment in the stables and more stable supply of food [10]. In Holland, the anophelines were observed to differ from those in malarious areas through earlier hibernation and staying inactive during the malaria transmission season, which was thought to have limited the potential of transmission [14]. In the UK, changes in agriculture and land drainage, anopheline biting habits, improvements in health and nutritional status of the human population, as well as changes in housing and ventilation are all thought to have contributed to the disappearance of malaria [15]. Moreover, in Finland, rather than temperature and vector behaviour changes, social changes such as land consolidation, decreasing household size and improved housing standards were thought to be the driving forces behind malaria decline [16].

In addition to those indirect drivers behind malaria recession in Europe, human interventions eventually played a part in freeing the continent from malaria transmission. Malaria vectors were identified at the end of the 19th century, and since then control measures aiming at breaking the transmission chain began to be used [17]. Following serious outbreaks of malaria after the First World War, anti-mosquito measures, primarily water drainage and larviciding, were carried out in different regions and countries across Europe [15, 17, 18], to destroy mosquito breeding sites and reduce populations. Meanwhile, the wider availability of quinine likely contributed to a decline in malaria parasites in humans [10, 19]. Later, with the advent of dichloro-diphenyl-trichloroethane (DDT) and its efficiency against *Anopheles* mosquitoes found, national anti-malarial activities as well as the Global Malaria Eradication Programme (GMEP) were launched in the 1940s and 1950s (Table 1), and DDT spraying was used intensively, together with drug distribution and other control measures. Unprecedented successes were seen. For example, in Italy, case numbers declined from around 50,000 in 1945 to less than 100 per year after a national anti-malarial campaign [9]. Similar declines were also seen in other European countries [9, 18] (Fig. 2). Although the GMEP failed to eradicate malaria globally, the continent of Europe became free of malaria transmission as part of its efforts.

**Table 1:** Dates of malaria decline and elimination in 31 European countries

Country	Decadal endpoint used to represent large malaria decline	Decadal endpoint of malaria elimination	Reported start year of malaria elimination programme	Year of last reported indigenous case	Year of WHO certification/estimate of malaria free status
Albania	1950	1970	1947	1966	2012
Austria	1920	1950			1963
Belarus	1950	1970	1951		2012
Belgium	1920	1950		1938	1963
Bosnia and Herzegovina	1950	1970	1947	1964	1973
Bulgaria	1950	1960	1947–1950	1957 (pf); 1960 (pv)	1965
Croatia	1950	1970	1947	1964	1973
Czech Republic	1950	1960			1963
Estonia	1950	1970	1951		2012
Finland	1920	1950		1954	1963
France	1920	1950		1950	2012
Germany	1920	1950		1950	1964
Greece	1960	1970	1946–1954	1973	2012
Hungary	1950	1960	1946	1962	1964
Italy	1950	1960	1930s; 1944–1945	1952 (pf); 1962 (pv)	1970
Latvia	1950	1970	1951		2012
Lithuania	1950	1970	1951		2012
Montenegro	1950	1970	1947	1964	1973
Netherlands	1950	1960	1946	1961	1970
Poland	1930	1960	After WW1; 1945	1955	1967
Portugal	1950	1960	1930; 1948	1958	1973
Moldova	1950	1970	1951		2012
Romania	1950	1970	1949	1962	1967
Russian Federation	1950	1970	1951		2012
Serbia	1950	1970	1947	1964	1973
Slovakia	1950	1960			1963
Slovenia	1950	1970	1947	1964	1973
Spain	1950	1960	1943	1962	1964
Macedonia	1950	1970	1947	1964	1973
Ukraine	1950	1970	1951		2012
UK	1920	1950	After WW1	1953	1963

The decadal endpoint used to represent the ‘large malaria decline’ was defined by Fig. 2 and literature. The decadal endpoint of malaria elimination was dependent on the year of last reported indigenous cases and that of WHO certification/estimate of malaria free status [9, 54]. The reported start year of the malaria elimination programme indicates the beginning time of anti-malarial programmes [9]. An extended version of the Table is provided in Additional file 1

A wide range of factors have therefore likely played a part in freeing Europe from endemic malaria transmission, but the contribution of each across the continent, and across time periods has never been simultaneously and quantitatively assessed. Moreover, the similarities in these factors between present-day malaria-elimination countries and those exhibited by European countries at the time of elimination have yet to be examined to assess whether commonalities in potential driving factors exist. Here, a range of spatial datasets representing climatic, land use and socio-economic factors thought to be associated with the decline of malaria in Europe were assembled, and integrated with historical malaria distribution maps to quantify changes and differences across the continent before, during and after malaria elimination. The differences and similarities that exist between current eliminating countries [4] and historical Europe in terms of candidate driving factors were also examined.

## Methods

Data on the spatiotemporal history of malaria in Europe, including historical distribution maps, reported case numbers and dates of control/elimination were assembled, together with spatial datasets depicting factors that have been linked to the decline of malaria in Europe. These datasets were integrated, and simple statistical tests were undertaken to assess the strength of relationships between candidate drivers and malaria declines, as well as similarities to conditions in present-day malaria-elimination countries [4].

### Definition of malaria decline and elimination

The continent of Europe is today composed of 50 sovereign countries. Some were originally malaria-free, some had eliminated malaria prior to the start of 20th century and some are especially small in area, but 31 countries eliminated endemic transmission between 1900 and 1975. These 31 European countries were therefore the focus of analyses undertaken here (Table 1). Despite some outbreaks related to major wars, which were mainly caused by the movement of refugees and returned soldiers [20], malaria in Europe had been declining from 1900 to 1975 [9] (Fig. 2). Three decadal timepoints were defined to describe the elimination timeline of each country. The year of 1900 was used as the baseline from which comparisons were made. Following that, a decadal timepoint was defined for each country for when malaria cases were at their greatest rate of decline (Fig. 2; Table 1). Finally, a decadal timepoint for malaria elimination was defined for each country. These were based on two timepoints: the year of malaria elimination certified by the WHO and the time of the last indigenous case reported in the individual country (Table 1).

### Data on candidate driving factors

Nine factors (Table 2) that describe climate conditions, socio-economic development and land use changes were selected as the candidate driving factors of malaria elimination here. The selection criteria were based on historical and current studies that explore the reasons behind malaria transmission and declines seen, as well as the availability of datasets. Climate changes, especially in temperature, are often considered to have an effect on malaria transmission [2, 21–25]. Temperature influences mosquito distribution, feeding intervals and lifespan, as well as the rate of parasite multiplication in female mosquitoes [25–27]. Moreover, the frequency of frost days can provide an indicator of the limits of survival of *Anopheles* mosquitoes. The role of precipitation in promoting malaria transmission is mainly through the creation of larval sites [28, 29], but also precipitation can increase atmospheric humidity, which affects the internal water balance of mosquitoes and thus reduces their longevity [30].

**Table 2:** Details of candidate malaria elimination driver variables data available

Variable	Format	Unit	Spatial resolution	Temporal resolution	Temporal frame	Sources
Daily mean temperature	ASCII	Degree (°C)	0.5°	Monthly	1901 ~ 2009	CRU-TS v3.10
Perception	ASCII	mm	0.5°	Monthly	1901 ~ 2009	CRU-TS v3.10
Frost day frequency	ASCII	Days	0.5°	Monthly	1901 ~ 2009	CRU-TS v3.10
GDP per capita	Aggregated	Fixed PPP$	National	Yearly	1800 ~ 2013	Gapminder
Life expectancy	Aggregated	Years	National	Yearly	1800 ~ 2013	Gapminder
Population density	Raster	People/grid	5 min	Decade	1900 ~ 2000	HYDE 3.1
Urban population density	Raster	People/grid	5 min	Decade	1900 ~ 2000	HYDE 3.1
Cropland	Raster	km^2^/grid	5 min	Decade	1900 ~ 2000	HYDE 3.1
Grassland	Raster	km^2^/grid	5 min	Decade	1900 ~ 2000	HYDE 3.1

Economic development (represented by gross domestic product (GDP) per capita here) is linked with general levels of poverty, which have a close association with malaria prevalence [31–33]. Individuals with more wealth tend to have better nutrition, living environments and healthcare access than those in poverty, providing barriers to the establishment and maintenance of malaria transmission. Moreover, wealthy countries generally have resources available to operate strong health systems and invest in tackling malaria, which is a vital factor to successful elimination programmes [34, 35]. Thus to some extent, the level of economic development can be a reflection of the strength of past control measures, since these remain difficult to quantify directly, especially between countries. Life expectancy provides a measure of the overall health of the population and the strength of the health system. Delivering effective curative treatments to infected cases rapidly reduces the parasites reservoir, and thus results in declining transmission rates. While urbanization has been widely shown to have a negative effect on malaria transmission because of fewer breeding sites in cities, improved housing and access to healthcare facilities, among other factors [36–38]. Finally, land use changes in terms of changing areas of cropland and grassland reflect changing agricultural practises, such as the rise in domestic animal numbers, and the draining of marshlands for cropping, which have both been implicated in driving malaria elimination in Europe [10, 15, 17, 39]. These data sources are described below and in Table 2.

Gridded monthly climate datasets for the 1901–2014 were downloaded from the Climatic Research Unit (CRU) TS (time-series) database [40]. The construction of these datasets is based on an archive of data collected from more than 4000 weather stations all over the world. Three metrics describing key climatic indicators were analysed here: daily mean temperature, frost day frequency and precipitation.

National level datasets on GDP per capita and life expectancy across 1800–2013 were obtained from Gapminder [41]. The GDP data were based on fixed 2011 prices, adjusted for purchasing power parities (PPPs) in international dollars [42]. Urbanization and land use data were obtained from the History Database of the Global Environment (HYDE) 3.1 [43]. Gridded layers of rural/urban population, urban population, cropland and grassland for each decade of the 1900–2000 period at 5 min spatial resolution were obtained.

### Data preparation

The gridded climate, land cover and urbanization datasets were aggregated to national levels for consistency with the GDP and life expectancy data. The monthly climate data were converted to five-year averages to remove noise and provide a picture of general climatic conditions. The urban/rural, urban population, cropland and grassland layers were aggregated to percentages for each country. Finally, from the complete set of national-level datasets, the per-country values for the three timepoints of 1900, the per-country malaria decline periods and the per-country malaria elimination periods (Table 1) were extracted. Through the same procedure, those datasets were also prepared for the present day for 34 malaria-eliminating countries [4] (Mayotte was not analysed because of too small an area to be represented in the gridded datasets).

### Data analyses

The changing patterns of candidate driving factors were explored in relation to the declines of malaria in Europe. Firstly, for those 31 European countries that eliminated malaria between 1900 and 1975, the candidate driving factor variables were compared between 1900 and the per-country malaria decline periods, followed by comparisons between the per-country malaria decline periods and the per-country malaria elimination dates. Following previous similar analyses [38], Wilcoxon signed-rank tests were applied to assess the significance of differences between two paired groups, without the assumption that the data were normally distributed. Following this, the similarities between Europe at the time of malaria decline and elimination, and the conditions in current elimination countries, were examined. As in previous similar studies [38], Mann–Whitney tests were used for the two non-paired groups. The data analyses and visualization was carried out in R [44].

## Results

### Comparisons in Europe

**Fig. 3:**
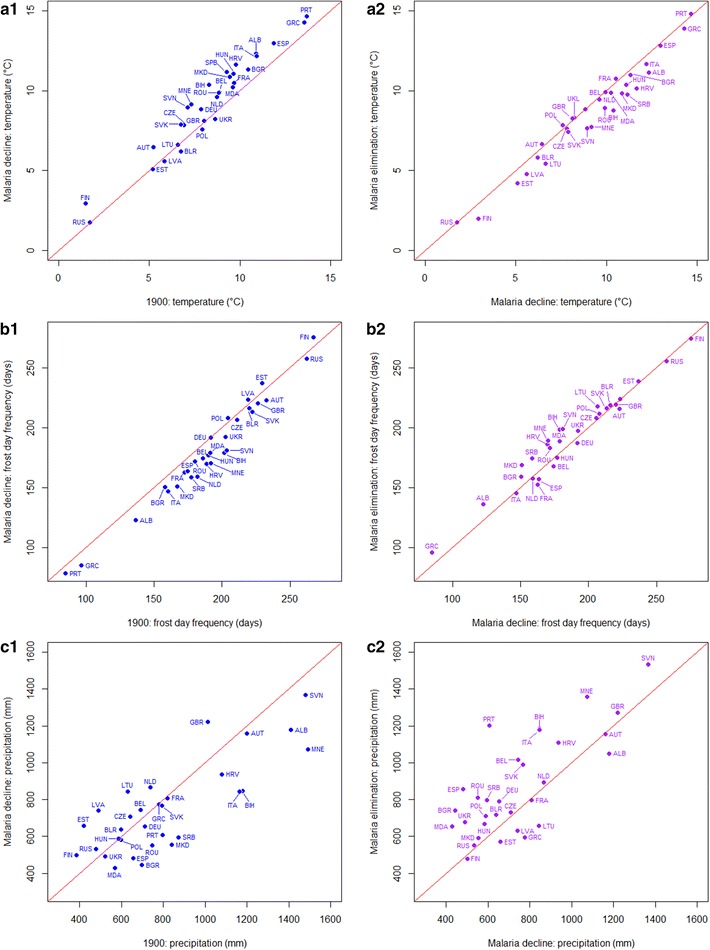
*Scatterplots* showing changes in temperature, frost day frequency and precipitation in Europe from 1900 to the per-country malaria decline periods (*blue*) and that from the per-country periods of malaria decline to elimination (*purple*). A *one-to-one line* is shown on each plot to aid interpretation. The ISO country abbreviation is used on the scatterplots for country names (http://www.nationsonline.org/oneworld/country_code_list.htm)

Changes in climate were not consistent with the declines of malaria seen in Europe (Fig. 3, Table 3), a similar result to that found elsewhere [2]. From 1900 to the time of malaria decline, most countries showed increasing temperatures (Wilcoxon test: z = −4.2916, p < 0.0001) and corresponding decreases in frost day frequency (Wilcoxon test: z = 4.2916, p < 0.0001). However, from the period of greatest decline of malaria to elimination, a trend of lower temperature (Wilcoxon test: z = 3.8508, p < 0.0001) and higher frost day frequency (Wilcoxon test: z = −2.2144, p < 0.05) was seen. For precipitation, despite substantial fluctuations by country, no overall significant difference (Wilcoxon test: z = 1.7049, p = 0.09) from 1900 to the decline of malaria was seen; while in the latter time period from greatest malaria decline to elimination, a significant increase (Wilcoxon test: z = −3.253, p < 0.001) in precipitation was found.

**Table 3:** Results of Wilcoxon tests comparing available candidate driver variables in 1900 and at the per-country period of malaria decline, as well as for per-country malaria decline and elimination

Variable	1900 vs malaria decline		Malaria decline vs elimination	
	z	p value	z	p value
Temperature	−4.2916	<0.0001	3.8508	<0.0001
Frost day frequency	4.2916	<0.0001	−2.2144	<0.05
Precipitation	1.7049	0.09	−3.253	<0.001
GDP	−4.4092	<0.0001	−4.8599	<0.0001
Life expectancy	−4.8599	<0.0001	−4.8599	<0.0001
Percentage urban population	−4.8599	<0.0001	−4.8599	<0.0001
Percentage urban area	−2.9102	<0.01	−4.8599	<0.0001
Percentage cropland	−4.1153	<0.0001	−4.5268	<0.0001
Percentage grassland	−3.0963	<0.01	−2.7043	<0.01

**Fig. 4:**
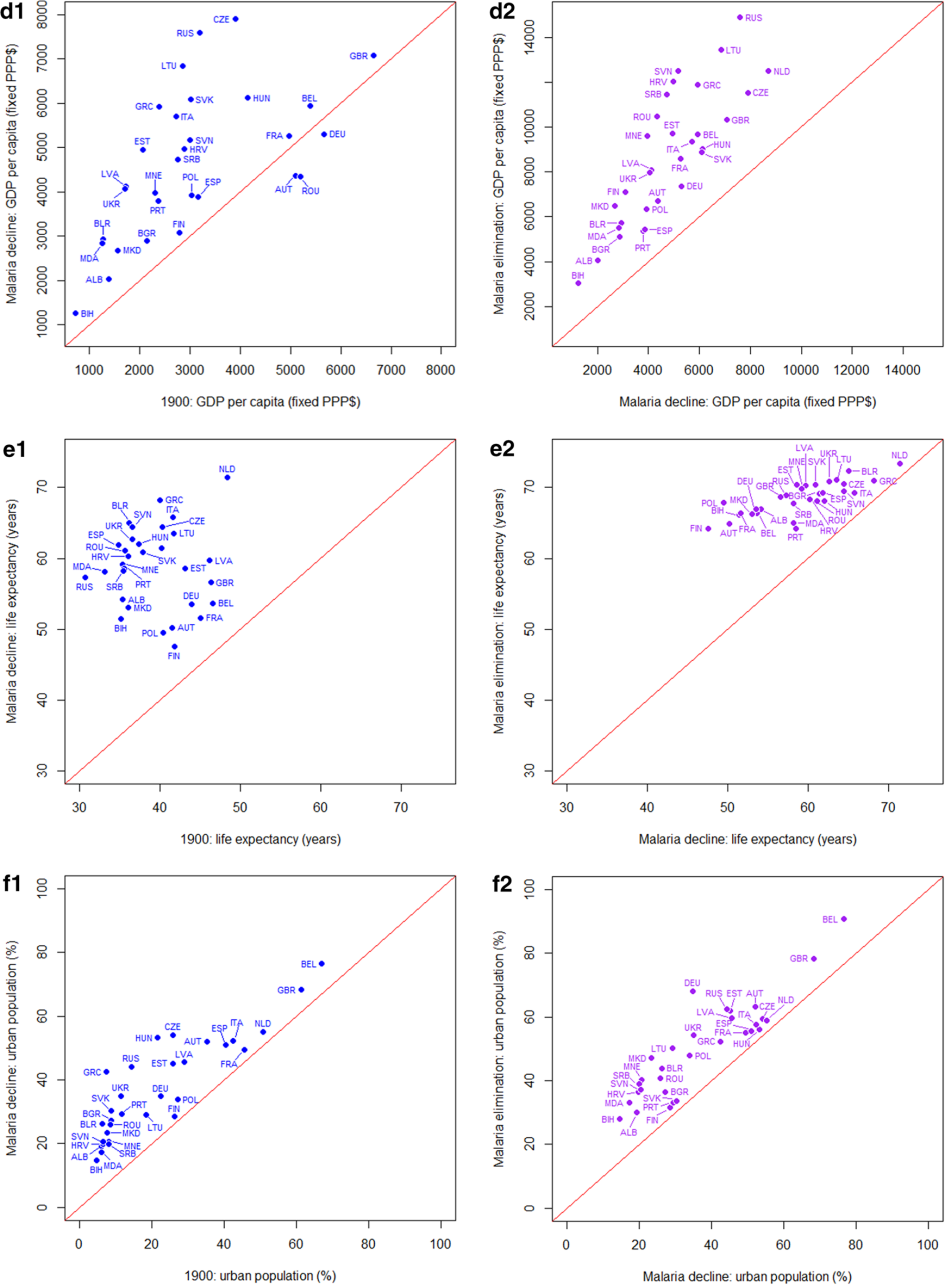
*Scatterplots* showing changes in GDP per capita, life expectancy, urban area and population from 1900 to the per-country malaria decline periods and from the per-country malaria decline periods to elimination. As the percentages of urban area in Belgium were far higher (30.47, 42.10 and 68.09 % at the time of 1900, malaria decline and elimination) than other countries, to reveal the general trend in Europe, data for Belgium were not shown here. A *one-to-one line* is shown on each plot to aid interpretation. The ISO country abbreviation is used on the scatterplots for country names (http://www.nationsonline.org/oneworld/country_code_list.htm)

GDP, life expectancy and proportion of population urbanized all showed consistent increases concurrent with the reduction of malaria in Europe (Fig. 4, Table 3). From 1900 to the period of malaria decline for each country, increases (Wilcoxon test: z = −4.4092, p < 0.0001) in GDP were seen. When elimination was achieved, all countries had higher GDP than at the time of greatest malaria decline (Wilcoxon test: z = −4.8599, p < 0.0001). Further, life expectancy had been continuously increasing during both the periods of malaria decline (Wilcoxon test: z = −4.8599, p < 0.0001) and elimination (Wilcoxon test: z = −4.8599, p < 0.0001). At the time elimination was achieved (which differed by country, Table 1), life expectancy in different countries was surprisingly similar. In addition, the proportions of population living in urban areas showed apparent increases during both the time periods examined (Wilcoxon test: from 1900 to malaria decline: z = −4.8599, p < 0.0001; from malaria decline to elimination: z = −4.8599, p < 0.0001).

**Fig. 5:**
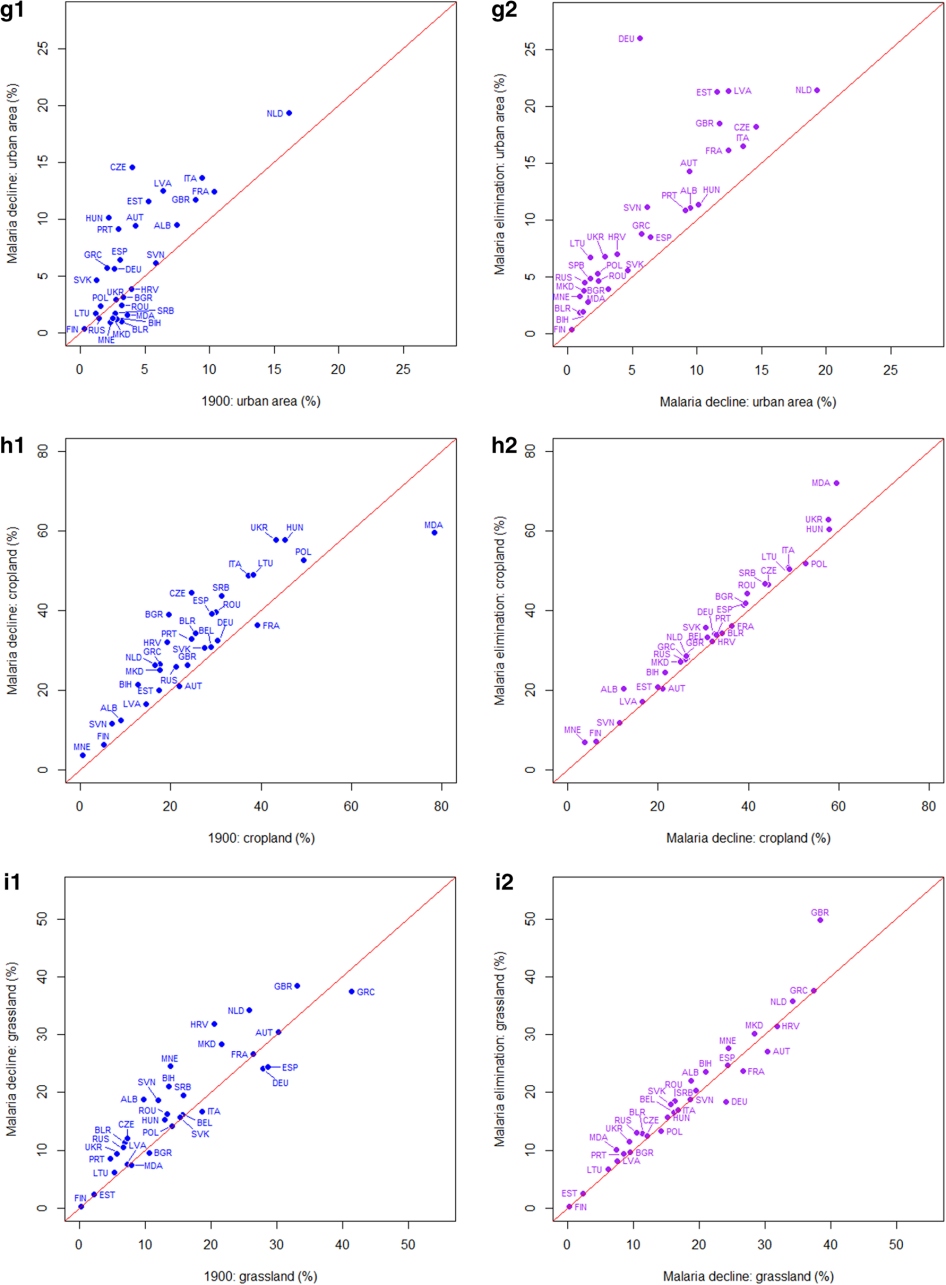
*Scatterplots* showing changes in the proportions of cropland and grassland from 1900 to the per-country malaria decline periods and from the per-country malaria decline periods to elimination in Europe. A *one-to-one line* is shown on each plot to aid interpretation. The ISO country abbreviation is used on the scatterplots for country names (http://www.nationsonline.org/oneworld/country_code_list.htm)

Unlike the socio-economic factors, which showed strong and consistent relationships with malaria declines and elimination, the changing land use data showed weaker linkages (Fig. 5; Table 3). Urbanized land area increased (Wilcoxon test: from 1900 to malaria decline: z = −2.9102, p < 0.01; from malaria decline to elimination: z = −4.8599, p < 0.0001). For cropland and grassland, great increases (Wilcoxon test: cropland: z = −4.1153, p < 0.01; grassland: z = −3.0963, p < 0.01) occurred during the periods of 1900 to the decline of malaria. But in the later time period, although the proportions of cropland and grassland continued to increase (Wilcoxon test: cropland: z = −4.5268, p < 0.01; grassland: z = −2.7043, p < 0.01), the magnitudes of these increases were much smaller.

### Comparisons with present-day countries aiming for elimination

**Fig. 6:**
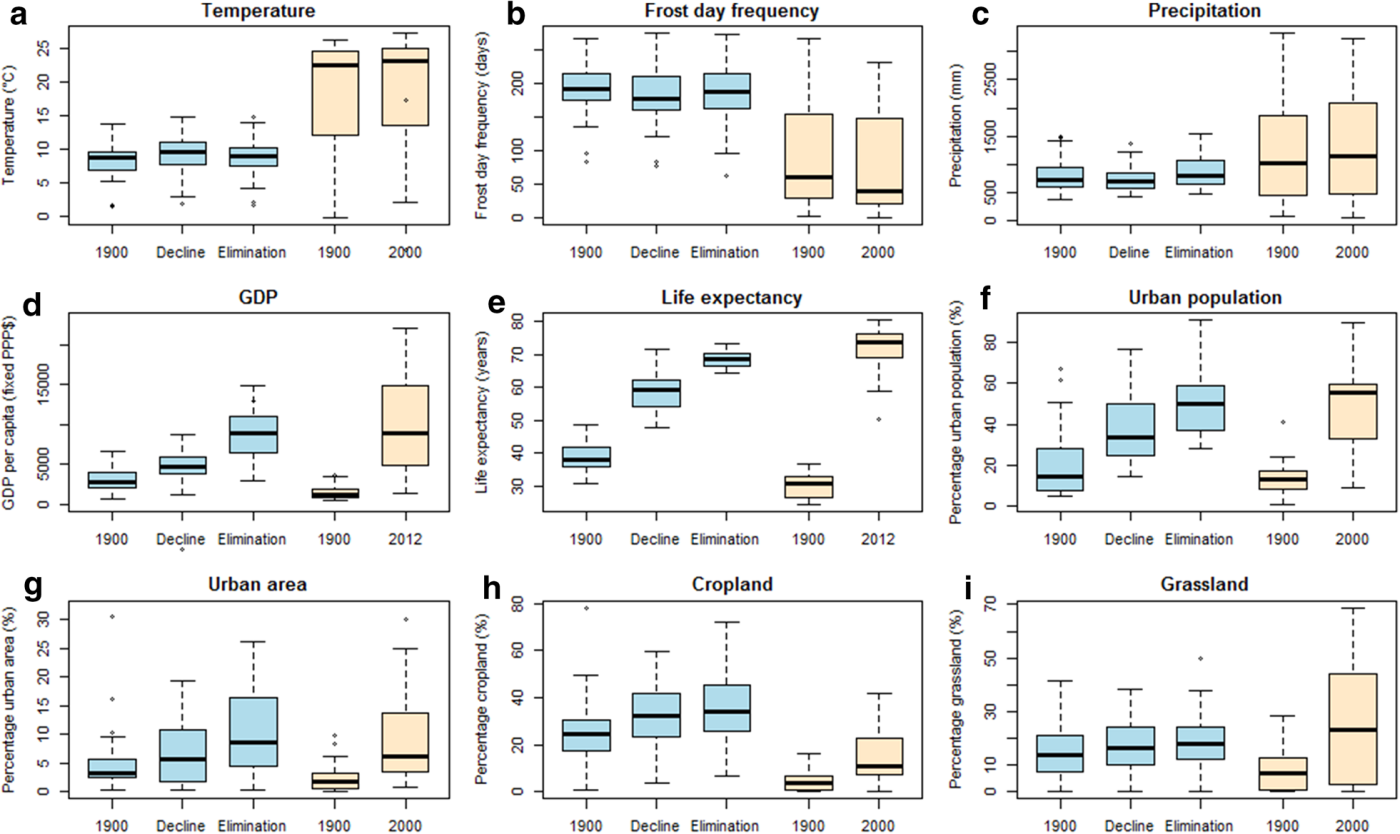
*Boxplots* comparing available candidate elimination driver variables in Europe and for eliminating countries over the last century. Variables in Europe are shown for the years of 1900, per-country malaria decline periods and per-country elimination years, with the same sources as Figs. 3, 4 and 5. In eliminating countries, variables for 1900 and 2000 are shown, except for GDP and life expectancy, which are presented for 2013 due to data availability

The 34 countries with current national goals of malaria elimination [4], hereafter referred to as ‘eliminating countries’, were compared with European countries in terms of the candidate driving factors at the three country-specific timepoints of malaria elimination (1900, period of greatest malaria decline, time of malaria elimination). Figure 6 and Table 4 summarize the similarities in candidate driving factors between those today in current elimination countries and those in European countries at the times of greatest malaria decline and elimination. Results of statistical tests for the comparisons between European and eliminating countries in 1900 as well as those for eliminating countries in 1900 and the present day are provided in Additional file 2.

**Table 4:** Results of Mann–Whitney tests comparing available candidate elimination driver variables in Europe at the time of malaria decline and elimination versus those for current malaria eliminating countries in the present day

Variable	Europe at the time of malaria decline vs eliminating countries in the present day		Europe at the time of malaria elimination vs eliminating countries in the present day	
	z	p value	z	p value
Temperature	−5.4769	<0.0001	−5.6214	<0.0001
Frost day frequency	5.1485	<0.0001	5.2273	<0.0001
Precipitation	−2.5086	<0.05	−2.0095	<0.05
GDP	−3.6775	<0.001	−0.52536	0.6062
Life expectancy	−5.9633	<0.0001	−3.7304	<0.001
Percentage urban population	−2.4167	<0.05	0.27581	0.7892
Percentage urban area	−1.3659	0.1752	0.6567	0.5181
Percentage cropland	4.3999	<0.0001	4.7677	<0.0001
Percentage grassland	−0.26268	0.7992	−0.15761	0.8808

In terms of climatic conditions, countries that currently have elimination goals, which are generally located in lower latitudes than Europe, unsurprisingly have higher temperatures and lower frost day frequency than conditions in Europe at the times of greatest malaria decline and elimination. The annual precipitation amount in eliminating countries today is slightly higher as well, but with a wide range.

More interestingly, it appears that the current eliminating countries have arrived at the same or even higher levels in terms of socio-economic metrics than European countries at the time that malaria was in greatest decline and at the time of elimination. Present-day elimination countries have generally higher levels of GDP than those in Europe at the time of malaria decline and at the same level as European countries at the time that malaria was eliminated. In terms of life expectancy, people in present-day eliminating countries generally have higher life expectancies than in Europe both at the time of greatest malaria decline and at elimination. Moreover, for today’s elimination countries, the proportions of their populations residing in urban areas are significantly higher overall than the levels in Europe when substantial malaria declines were seen and at the same level as when elimination was achieved. In terms of land use types, present-day elimination countries overall show similar percentages of urban area and grassland to Europe at both the time of greatest malaria decline and at elimination, but lower percentages of cropland.

Analyses were stratified by continent to explore regional variations (Table 5). Unsurprisingly, all eliminating countries across the three continents have higher mean temperatures and lower frost day frequencies than in Europe during malaria decline and elimination. For precipitation, apart from similar levels in Asia, the elimination countries in both the Americas and Africa had lower precipitation than those in Europe at the time of malaria decline and elimination. For the socio-economic indicators, no continental variations were seen, with today’s elimination countries showing similar levels of GDP and urban population proportions to Europe when elimination was achieved. For life expectancy, current elimination countries in the Americas and Asia display significantly higher life expectancies than Europe at the time of elimination, and similar life expectancies are seen in the African elimination countries. Finally, higher proportions of grassland than the European countries at the time of elimination were seen in the elimination countries of the Americas, and similar levels in African and Asian elimination countries.

**Table 5:** Results of Mann–Whitney tests comparing available candidate elimination driver variables in Europe at the time of malaria elimination versus current eliminating countries by continent

Variable	Americas		Africa		Asia	
	z	p value	z	p value	z	p value
Temperature	−4.7056	<0.0001	−4.0856	<0.0001	−3.1282	<0.01
Frost day frequency	4.7056	<0.0001	4.0103	<0.0001	2.4955	<0.05
Precipitation	4.7056	<0.0001	2.1275	<0.05	−1.113	0.2742
GDP	−0.39466	0.7087	−0.35773	0.7403	−1.0896	0.2846
Life expectancy	−4.311	<0.0001	1.0732	0.2989	−4.6516	<0.0001
Percentage urban population	−1.3965	0.1698	0.39538	0.7126	0.59752	0.5621
Percentage urban area	−1.3358	0.1898	0.50835	0.6317	1.4645	0.1477
Percentage cropland	3.218	<0.001	3.822	<0.0001	2.9642	<0.01
Percentage grassland	−2.1555	<0.05	−1.0355	0.3167	1.3474	0.1838

## Discussion

The decline and elimination of malaria were likely the result of a combination of factors that affect the process of malaria transmission [10, 39]. Here, it was found that socio-economic changes such as increases in GDP, life expectancy and urbanization were significantly correlated with the decline and elimination of malaria in Europe. Land use changes also showed associations, but were not as strong. Further, changes in climate were unlikely to have played a role in malaria reduction and elimination, but the inherent climate conditions likely contributed to the low receptivity of malaria in Europe.

If the socio-economic and land use factors found to be associated with malaria declines in European countries were playing a role in both driving the decline and elimination, and maintaining malaria-free status, then this offers great hope to modern-day malaria elimination countries. Having reached similar or high socio-economic levels as those seen in Europe at the time of greatest malaria declines and elimination, and with extensive control efforts in place, today’s elimination countries may be well placed to achieve and sustain malaria elimination. This hope should of course be tempered by the different ecological landscapes that exist in today’s elimination countries that are more receptive to transmission, as well as rising human mobility, likely resulting in recurring introductions from different regions in the world. Nevertheless, with large declines in prevalence over the past decade driven in part by intervention scale-up [45], it seems likely that rising economic development, urbanization, improved population health, and changing land use practices have also played a role.

Where significant differences from Europe at the time of malaria elimination exist, they are in climatic conditions. Higher temperatures, increased precipitation and fewer frost days all suit malaria transmission, making present-day elimination countries more receptive to transmission, and thus more of a challenge in terms of achieving elimination than was the case in Europe [46, 47]. Nevertheless, many European countries remain receptive to transmission today, but outbreaks rarely occur, despite significant numbers of imported cases [48]. A combination of factors is likely behind these ‘sticky’ states [5, 6], and some of these are related to economic development, land use and health systems, which have made elimination sustainable. With today’s elimination countries approaching or exceeding the levels that European countries were at when elimination was achieved, it seems feasible that they are moving towards similar sticky elimination states.

The results here point towards socio-economic and land use related factors being at least partial drivers of malaria decline and elimination in Europe, but many other factors likely played a part, for which historical consistent data are lacking. These include the strengthening of surveillance and health systems, changing lifestyles, household construction, and differing *Anopheles* species compositions. The candidate factors examined here are all related in some way to these differing drivers, but without historical data, specific interactions and associations cannot be assessed. Moreover, the quality and completeness of the datasets used here likely declines back in time towards the start of the 20th century, increasing uncertainties in associations found. The clear and consistent relationships found however suggest that these do not have a major impact.

As the international community focuses efforts on shrinking the malaria map through continued intervention [49], the impact of non-control factors on transmission remains an area of research. Factors such as urbanization [38], housing [50] and socio-economic development [51], have all been shown to contribute to declines in malaria transmission with sustained impact. These likely played a significant role in malaria declines and elimination in Europe, and will continue to contribute elsewhere, offering hope that the recent gains made against malaria can be maintained.

## Additional files

10.1186/s12936-016-1175-z Details on reported years for malaria elimination for European countries.
10.1186/s12936-016-1175-z Results of statistical tests examining the differences in climatic, land use and demographic variables between European countries and those focused on eliminating the disease at the time of writing.
